# The Diagnostic Potential of Axon Excitability Is Consistent Across Hand Muscles in Amyotrophic Lateral Sclerosis

**DOI:** 10.1002/mus.70239

**Published:** 2026-04-11

**Authors:** Alana K. O. Hodgson, Christopher J. McDermott, Pamela J. Shaw, James J. P. Alix

**Affiliations:** ^1^ Sheffield Institute for Translational Neuroscience University of Sheffield Sheffield UK; ^2^ Neuroscience Institute University of Sheffield Sheffield UK; ^3^ NIHR Sheffield Biomedical Research Centre, Sheffield Teaching Hospitals NHS Foundation Trust Sheffield UK

**Keywords:** amyotrophic lateral sclerosis, diagnosis, motor unit number, nerve excitability

## Abstract

**Introduction/Aims:**

Recent work suggests that nerve excitability testing has diagnostic potential in amyotrophic lateral sclerosis (ALS). The diagnostic performance of nerve excitability across hand muscles is currently unknown. This study aimed to assess if muscles of the so‐called split hand (abductor pollicis brevis [APB], first dorsal interosseous [FDI], and abductor digiti minimi [ADM]) manifest differences in diagnostic performance.

**Methods:**

We prospectively recruited 60 consecutive patients investigated for ALS. Nerve excitability, motor unit number and size (MScanFit), needle electromyography (EMG), and standard clinical data were collected. ALS and non‐ALS groups were compared using *t* tests, area under receiver operating characteristic curves (AUROC), and multivariate modeling.

**Results:**

Forty‐eight patients completed testing of all three muscles, 25 were diagnosed with ALS. The most prominent nerve excitability changes were in superexcitability (APB *p* = 0.001, FDI *p* = 0.0001, ADM *p* = 0.002). Diagnostic performance with superexcitability was similar across the three muscles (*p* > 0.05). Reductions in motor unit number were observed in ALS patients. Changes in excitability were evident without loss of motor units, most frequently in APB (40% of recordings). Improvements to the AUROC were obtained using combined excitability/motor unit parameters from APB/FDI (AUROC 0.97, *p* = 0.01 vs. FDI superexcitability alone). Combined excitability and motor unit modeling outperformed detection of EMG abnormalities.

**Discussion:**

Disturbances to nerve excitability are similar across the split hand muscles at the time of ALS diagnosis. These occurred prior to motor unit loss and traditional EMG changes. Combining excitability and motor unit parameters in the lateral hand can identify early pathology and potentially lead to earlier diagnosis.

## 1 Introduction

Altered excitability, typically hyperexcitability, is considered a key step in the pathophysiology of amyotrophic lateral sclerosis (ALS), driving both upper motor neuron (UMN) and lower motor neuron (LMN) degeneration [1, 2, 3]. In the brain, threshold tracked transcranial magnetic stimulation (TMS) studies have shown that motor cortex hyperexcitability can be identified subclinically and contribute to an earlier diagnosis [4, 5]. Work in peripheral nerves has demonstrated altered excitability before and following a loss in compound muscle action potential (CMAP) amplitude [6, 7].

The potential of nerve excitability testing (NET) to identify early LMN abnormalities and contribute to diagnosis is less well studied. A recent pioneering report began to address this by demonstrating the potential of NET, in combination with the motor unit number estimation technique MScanFit, to contribute to the diagnosis of ALS [8]. In this, recordings were performed on a single nerve/muscle (median nerve/abductor pollicis brevis [APB]) and the combined excitability/motor unit regression model identified abnormalities in patients who were otherwise Gold Coast criteria (GCC) negative [9].

The choice of APB by Stikvoort García et al. was likely driven by the common involvement of this muscle at the time of diagnosis as part of the split hand phenomenon [10]. APB was also the subject of earlier axon excitability studies detailing the differences between patients with ALS, healthy controls and a range of other conditions [11]. The other muscles of the split hand (first dorsal interosseous [FDI] and abductor digiti minimi [ADM]) have also been studied in ALS using excitability techniques. These have revealed potentially important differences in biophysical parameters, suggesting a higher physiological level of excitability in APB/FDI axons relative to ADM [12, 13, 14, 15]. However, some authors have not found such a split hand distribution [16].

In the present study we sought to assess if excitability measurements from the three muscles (APB/FDI/ADM) have differences in diagnostic performance. We also explored the relationship between excitability measures and motor unit number/EMG.

## 2 Methods

### 2.1 Participants

Consecutive patients were prospectively recruited into the study in keeping with the standards for reporting of diagnostic accuracy (STARD) criteria. Patients were recruited at the time of investigation into their symptoms (i.e., their first presentation to our clinic). Inclusion criteria included referral by a consultant neurologist for neurophysiological studies at the Royal Hallamshire Hospital, Sheffield, UK for suspected ALS/motor neuron disease (MND), age > 18 years and the capacity to provide informed consent. Exclusion criteria included any skin condition over the electrode placement sites and any long‐term immobilization (> 3 months) caused by a condition other than the primary neurological disorder under investigation. In keeping with other studies ascertaining the diagnostic value of LMN tests in ALS [17], we did not exclude patients in whom there were coexistent neuropathies (entrapments or otherwise) as these are common differential diagnoses, both in our experience and in other centers [17, 18]. In addition, we did not exclude patients who had been recently commenced on riluzole. All patients provided written informed consent. The study was approved by a research ethics committee (23/NW/0107).

### 2.2 Clinical Assessment

A final diagnosis of ALS was made according to GCC [9]. For these patients, site of onset, disease duration, disease progression rate (48‐ALSFRS‐R/symptom duration in months [19, 20]) were collected.

### 2.3 Electrophysiological Testing

The most clinically affected side was studied, unless all responses were absent. If a CMAP was absent in a single muscle, recordings were still performed on the other muscles in that hand. If there was no asymmetry in disease (e.g., bulbar presentation with no limb symptoms, bilateral symptoms), the dominant hand was studied. Nerve excitability recordings were undertaken using the TROND protocol using QTRAC software (Institute of Neurology, London, UK) [21]. Skin temperature at the site of recording was maintained at > 32°C. Nerve stimulation was provided by a DS5 bipolar stimulator (Digitimer Ltd). 3 M Red Dot electrodes (Saint Paul, Minnesota, USA) were used for stimulation, recording electrodes were Ambu Neuroline 700 (Ballerup, Demark). Responses were recorded using a D440‐2 amplifier (Digitimer Ltd., Welwyn Garden City, UK) with an inline HumBug 50 Hz noise eliminator (Digitimer Ltd., Welwyn Garden City, UK) and a National Instruments data acquisition system (NI DAQ USB‐6341‐BNC, X series, National Instruments, Austin, Texas, USA). Electrodes remained in place for MScanFit recording. Motor unit number and the mean motor unit amplitude (μV) were reported. For all muscles the active electrode was placed over the muscle belly. For APB and FDI, the reference electrode was placed over the metacarpophalangeal joint of the first digit, for ADM the metacarpophalangeal joint of the fifth digit. For MScan, stimulation pulse width was 0.2 ms, unless supramaximal stimulation could not be achieved. In these instances a width of 0.3 ms was used. Recordings were otherwise performed as described previously [22, 23].

### 2.4 Data Analysis

A sample size calculation was performed, based on the most prominent excitability differences between patients with ALS and patients with other conditions, as reported in publications available at the time the planning of the study [6, 24]. Random numbers (*n* = 500) were generated for each of these populations and receiver operating curve (ROC) plots constructed. Sample size calculations for the estimate and limits of the area under the ROC (AUROC) ranges indicated a requirement for 54–78 patients. From this, a total sample size (ALS + not ALS) of 60 was chosen. ALS versus non‐ALS comparisons were undertaken with unpaired *t* tests and false discovery rate correction (Benjamini‐Hochberg technique). For this, related sections of excitability were grouped together (see [25]). ROCs for single parameters were constructed using univariate logistic regression. Multivariate regression was performed with feature selection using the Akaike's Information Criterion (AIC). Only parameters with statistically significant estimates were retained within the final model. Cut‐off values were obtained with the Youden index. Fishers exact test was used to assess differences in gender between the two groups. Correlation was assessed using the Pearson correlation coefficient. *p* < 0.05 was deemed statistically significant. Data presented are mean ± standard error of the mean unless otherwise stated. Analyses were performed using JMP Pro17.2.0 (SAS Institute Inc., Cary, NC, 1989–2023).

## 3 Results

**FIGURE 1:**
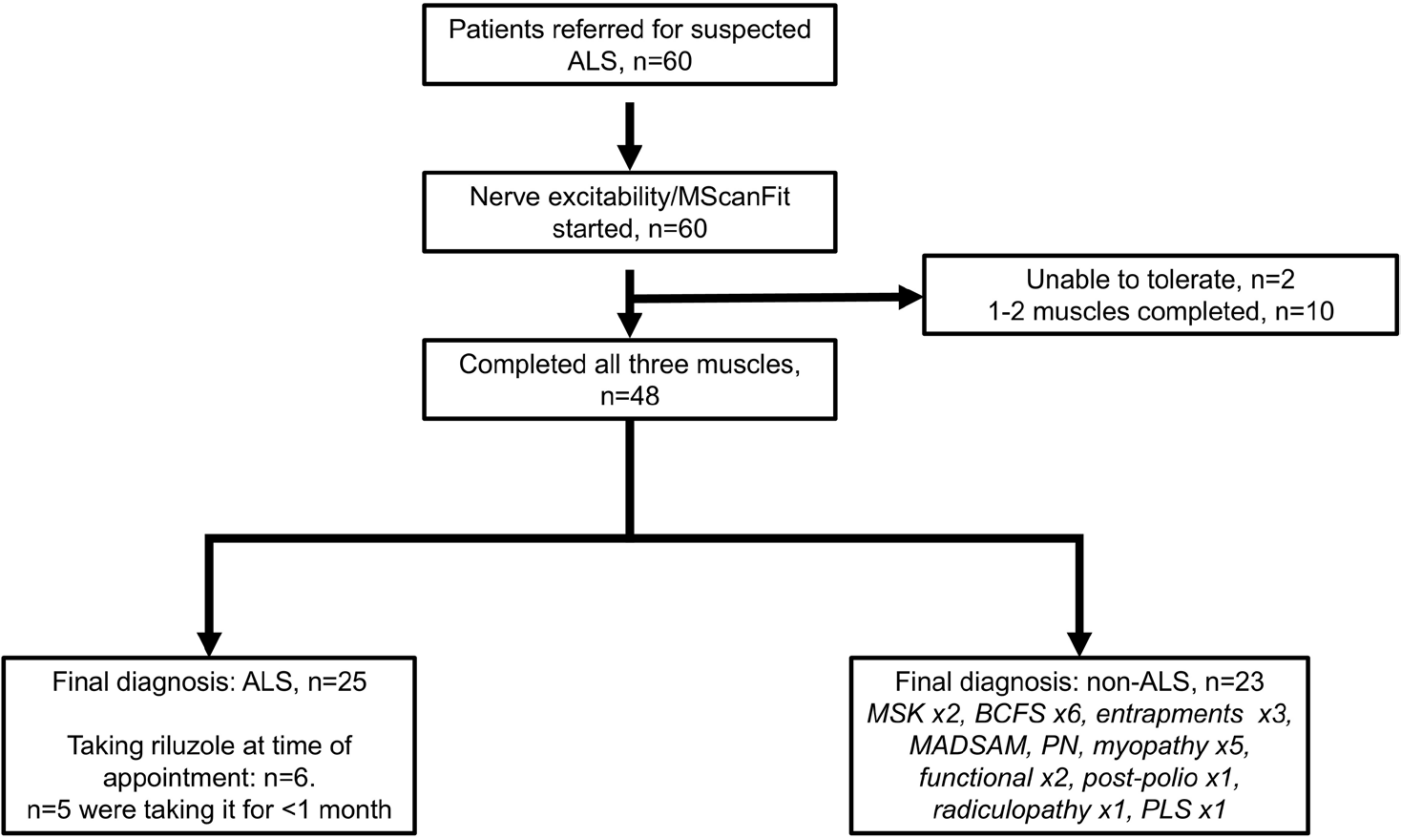
Study flow chart and patient characteristics. All patients were recruited at their first presentation to the clinic. MSK, musculoskeletal (arthritis); BCFS, benign cramp fasciculation syndrome; PLS, primary lateral sclerosis.

Sixty patients were recruited. Of these, *n* = 2 were unable to tolerate the recordings and no data of sufficient quality were collected. From the remaining *n* = 58, data from only 1–2 muscles were collected in *n* = 10 (due to small CMAP sizes); these patients were excluded from the final analyses. Thus, a total of 25 patients with ALS and 23 with other conditions were included (Figure 1). The mean age of the two groups was 61 years, with a higher proportion of males in both groups (Table 1). Of note, within the final diagnosis of the ALS group, *n* = 3 were initially diagnosed with primary lateral sclerosis, but transformed to ALS within 1 year and so were included within the ALS group. One further patient with no evidence of transformation was placed in the non‐ALS group (see discussion).

**TABLE 1:** The demographic and clinical details of the ALS and non‐ALS patient groups.

	ALS (*n* = 25)	Non‐ALS (*N* = 23)	*p* (statistical test)
Mean age in years (range)	61 (33–78)	61 (32–81)	*p* = 0.97 (*t* test)
Male:Female [*n* (%)]	16:9 (64:36)	18:5 (78:21)	*p* = 0.34 (Fisher's test)
Site of disease onset [*n* (%)]	Limb: 15 (60%) Bulbar: 10 (40%)		
Mean symptom duration (months, range)	14.5 (4–36)		
Mean disease progression rate (range)	0.89 (0.06–5.25)		
Gold Cost cervical EMG segment positive [*n* (%)]	18 (72%)		
Mean ALSFRS‐R score (range)	40 (27–46)		

Abbreviations: ALSFRS‐R, amyotrophic lateral sclerosis functional rating scale‐revised; EMG, electromyography.

**FIGURE 2:**
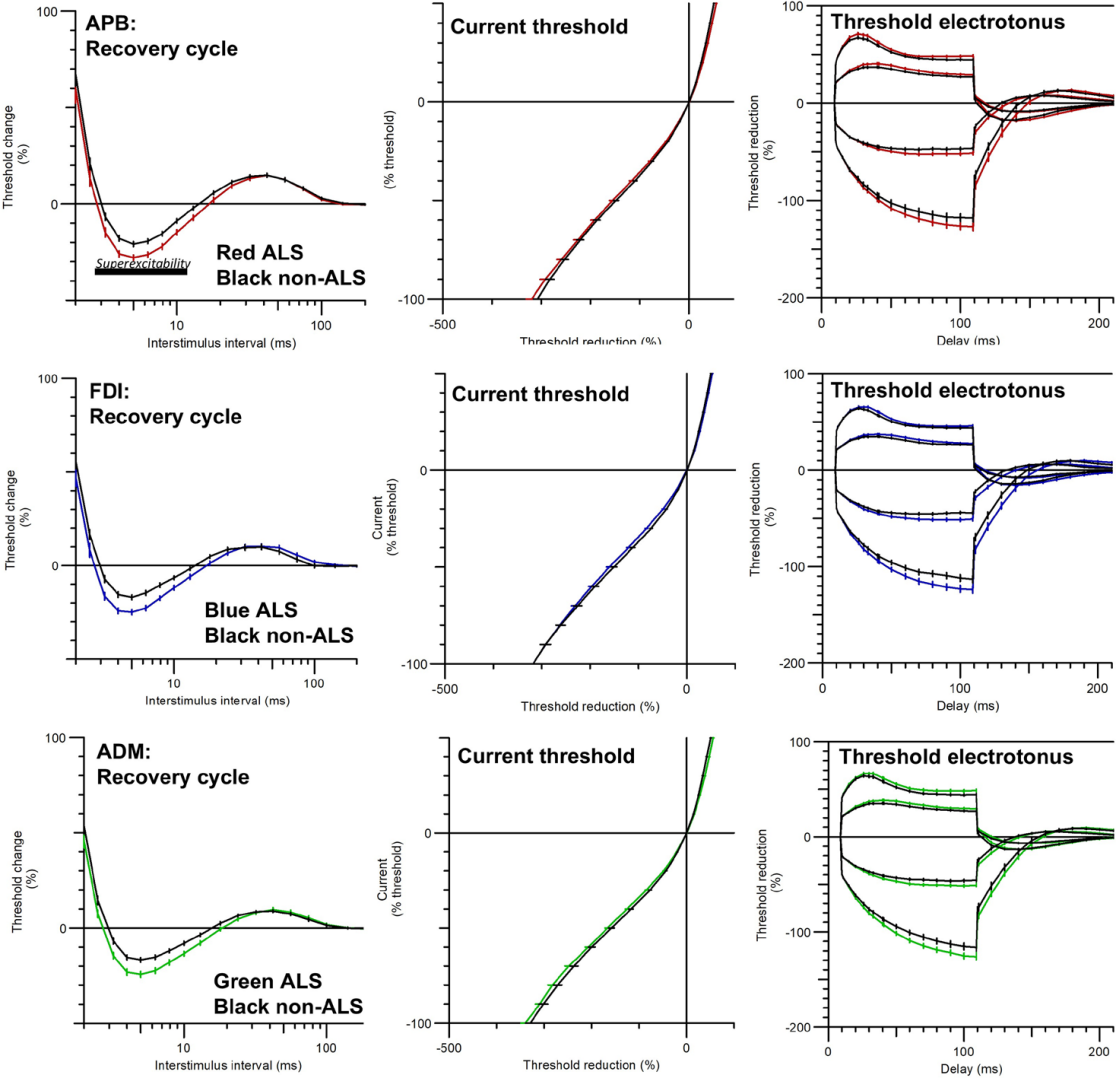
Excitability plots for APB, FDI, and ADM. Prominent differences in superexcitability (marked in the APB plot) were evident between the ALS and non‐ALS groups across all three muscles.

Examination of differences in nerve excitability demonstrated that patients with ALS had significantly different recovery cycles across all three muscles (Figure 2). The magnitude and period of superexcitability was greater in ALS patients and the relative refractory period was also shorter (see also Table S1). In the lateral hand muscles (i.e., APB and FDI), resting I/V slope differences were also evident (Table S1), however, there were no other significant differences after false discovery rate correction.

**FIGURE 3:**
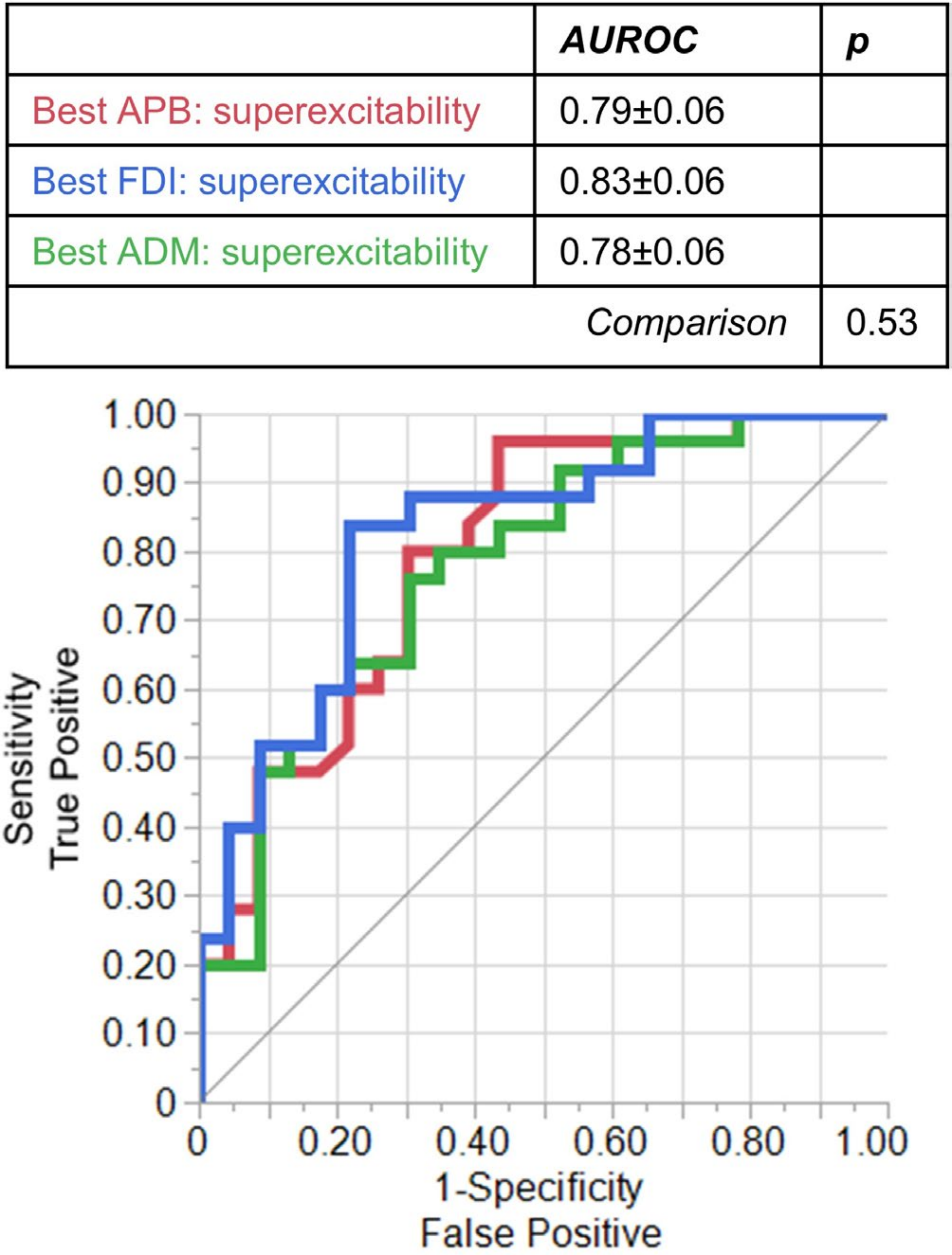
ROCs for superexcitability in each muscle. Superexcitability was the single excitability parameter with the highest diagnostic performance. Multivariate modeling was attempted but no additional parameters were selected. All three muscles demonstrated comparable diagnostic performance.

We next assessed the diagnostic potential of excitability testing in each of the three muscles (Figure 3). Multivariate modeling was attempted, but no parameters were retained by the AIC step‐wise regression beyond superexcitability. There was no significant difference between the AUROC for the three muscles (*p* = 0.53).

**FIGURE 4:**
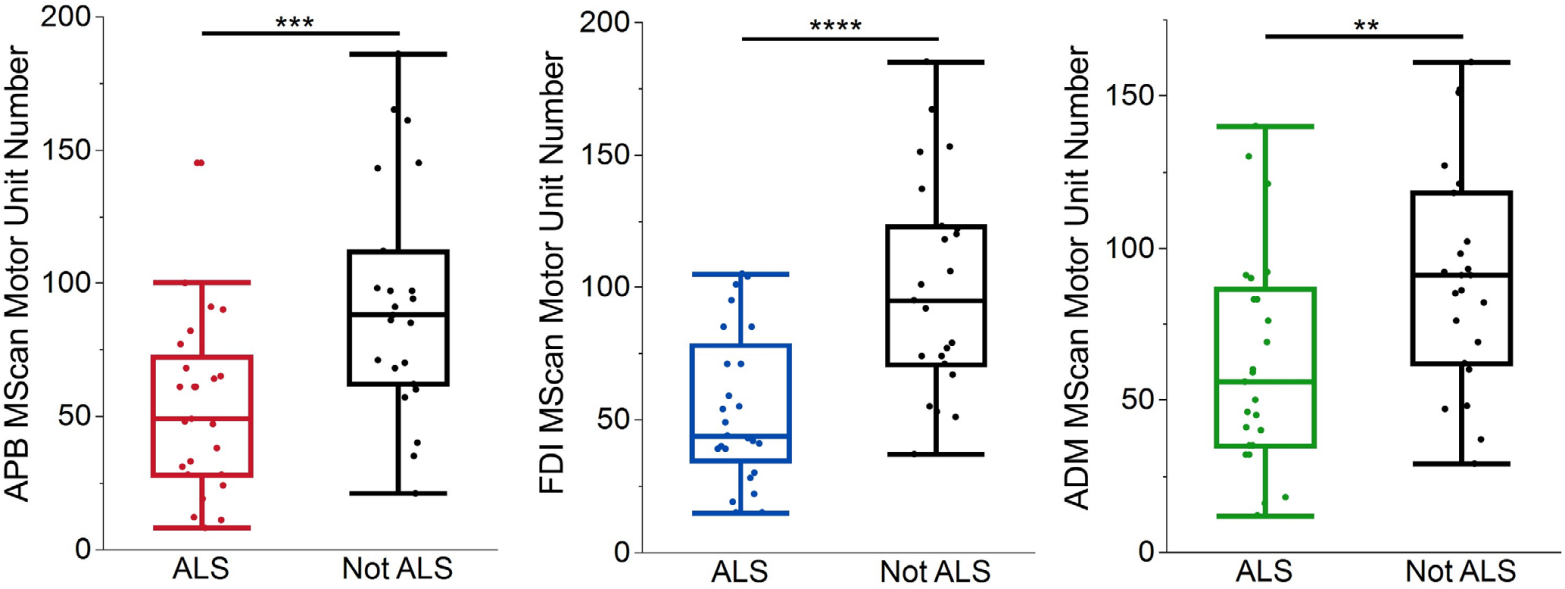
MScanFit derived motor unit number differences between ALS and non‐ALS patients. Lower motor unit numbers were evident in patients with ALS across all three muscles. **p < 0.01, ***p < 0.001, ****p < 0.0001.

In addition to nerve excitability, motor unit counts were obtained for each muscle. These demonstrated significant differences in motor unit number between ALS and non‐ALS patients (Figure 4). Motor unit size was only statistically different in APB and FDI (Table S2).

To assess if excitability was abnormal, with or without low motor unit numbers, in patients with ALS, we derived cut‐offs (Youden index) for motor unit number and superexcitability (Table 2). Applying these we found that low motor unit number and abnormal superexcitability was the most common observation (44%–52%, across the three muscles), followed by normal motor unit number but abnormal superexcitability (28%–40%). By contrast, it was rare for there to be low motor unit number without abnormal superexcitability (0%–12%). In addition, in ALS patients with/without electrophysiological evidence of the split hand phenomenon, there was no significant difference in superexcitability in any of the three muscles (Figure S1).

**TABLE 2:** Concordance between motor unit number and superexcitability.

	APB Superex. normal (cut off—19.7%)	APB Superex. abnormal
APB MU number normal (cut off 65)	2 (8%)	10 (40%)
APB MU number abnormal	0	13 (52%)

	FDI Superex. normal (cut off—18.8%)	FDI Superex. abnormal
FDI MU number normal (cut off 49)	3 (12%)	7 (28%)
FDI MU number abnormal	1 (4%)	13 (52%)

	ADM Superex. normal (cut off—18.6%)	ADM Superex. abnormal
ADM MU number normal (cut off 59)	3 (12%)	8 (32%)
ADM MU number abnormal	3 (12%)	11 (44%)

Abbreviations: ADM, abductor digiti minimi; APB, abductor pollicis brevis; FDI, first dorsal interosseous; MU, motor unit; Superex., superexcitability.

**FIGURE 5:**
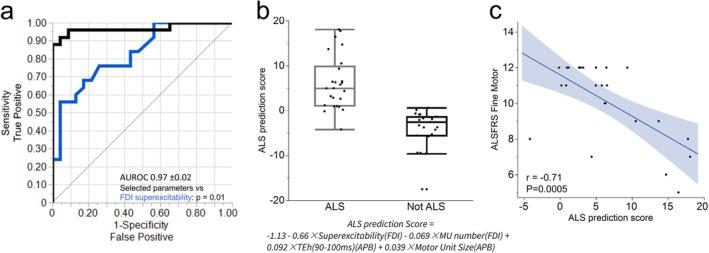
Combining excitability and motor unit parameters from the lateral hand muscles improves diagnostic performance and correlates with fine motor performance. (a) Stepwise feature selection using the AIC selected four parameters (FDI superexcitability, FDI motor unit number, APB TEh(90–100 ms) and APB mean motor unit size). This combination produced a greater AUROC than for FDI superexcitability (the best performing univariate model). (b) The coefficient from the logistic regression model can produce a prediction score (equation below graph). The further away from zero (in either direction), the more confident the prediction. (c) In patients with ALS, the ALS prediction score correlated with the fine motor subscore of the ASLFRS‐R, further evidencing that the model was capturing clinically relevant information.

We next assessed if a combination of excitability and MScanFit parameters across the three muscles could enhance the electrophysiological diagnostic performance. Using feature selection based on the AIC, two features were selected from FDI (motor unit number and superexcitability), and two different features were selected from APB (motor unit size and TEh[90–100]). This provided a significant improvement over the best univariate performance (*p* = 0.01, Figure 5a). The regression parameters are shown in Table S3. Utilizing the coefficient from this model, an ALS prediction score was computed (Figure 5b), which demonstrated a strong and significant correlation with the ALSFRS‐R fine motor subscore (Figure 5c).

Subsequently, we examined if the combined APB/FDI model was abnormal, or not, in the context of GCC electroclinical data (Table 3). While the most common finding was that of concordance (80% overall: 72% APB/FDI NET and GCC positive, 8% were negative on both), 20% of patients were positive on the APB/FDI NET model but negative as per the GCC. When considering the overall diagnosis, three patients (12%) would have achieved a quicker diagnosis if the model prediction score was applied at the time of investigation. We also examined if the combined motor unit/NET modeling was abnormal when compared to EMG at a single muscle level, using data from FDI (as the majority of patients had EMG of FDI, as well as motor unit/NET to this muscle). Concordance was again the most common finding (68%), followed by positive NET and negative EMG (24%, Table 3).

**TABLE 3:** Concordance between electroclinical GCC findings and the motor unit/nerve excitability regression model.

	Combined APB/FDI regression: not ALS	Combined APB/FDI regression: ALS
Cervical segment GCC NEGATIVE	2 (8%)	5 (20%)
Cervical segment GCC POSITIVE	0	18 (72%)

	FDI Superex. normal (cut off—18.8%)		FDI Superex. abnormal
FDI EMG: GCC NEGATIVE	1 (4%)		6 (24%)
FDI EMG: GCC POSITIVE	2 (8%)		16 (64%).

Abbreviations: ADM, abductor digiti minimi; APB, abductor pollicis brevis; EMG, electromyography; FDI, first dorsal interosseous; GCC, Gold Coast Criteria; Superex., superexcitability.

## 4 Discussion

The results show that there is no evidence of the split hand phenomenon in excitability testing, with all three muscles presenting similar diagnostic potential. However, motor unit number and size were differentially affected, and so combining excitability and motor unit parameters from the most affected muscles of the split hand conferred a higher diagnostic performance.

Nerve excitability has been undertaken extensively in ALS, but only one prior study has examined its diagnostic potential [8]. Our key findings agree with that study, namely that a combination of motor unit and nerve excitability parameters provides the best diagnostic performance and that abnormalities are seen even when the cervical segment is negative on GCC. This is despite some methodological differences, such as the exclusion of patients taking riluzole and patients with carpal tunnel syndrome by the previous study. While their rationale for excluding such patients was sound, our diagnostic performance did not decline as a result of including them. In addition, their multivariate modeling included parameters selected on the basis of previously published differences between ALS patients and healthy controls, while we took a data driven approach. We did try utilizing the combination proposed by Stikvoort Garcia et al., but not all of the parameter estimates of the model were significant, possibly as we had fewer patients in our cohort.

The recovery cycle, and in particular, superexcitability, was different when comparing ALS and non‐ALS cases. Superexcitability is consistently identified as an altered parameter in ALS [8, 11, 12, 26, 27]. The increase in superexcitability, together with other recovery cycle alterations, provides strong evidence for a reduction in K+ currents, most probably relating to impairment of fast paranodal/juxtaparanodal K+ channels [28]. This is supported by gene expression and immunohistochemical studies in spinal motor neurons from ALS patients [29, 30]. Our data would thus further support exploration of K channels as a therapeutic target [31].

Strength duration time constant (SDTC) is often reported as significantly altered in patients with ALS, typically comparing to healthy controls (e.g., [12, 26, 27]). However, SDTC has also been reported as normal [32], or only prolonged in the context of normal CMAP amplitudes [6]. Of relevance, Stikvoort Garcia et al. [8], did not see a group level difference in SDTC. Thus, it seems that this parameter is more variable in ALS populations than superexcitability. Of the other parameters identified in a recent meta‐analysis, resting I/V slope was abnormal in lateral hand muscles in our data, but depolarizing threshold electrotonus was not once the FDR had been applied.

Our data indicate that there is no evidence of an excitability‐based split hand at the time of an ALS diagnosis. Some prior reports found differences between lateral and medial hand muscles in patients with ALS [12] and healthy volunteers [13, 14, 15], leading to proposals that ion channel differences may underpin the split hand phenomenon. However, this has not been consistent [16, 32]. A possible explanation is that cortical mechanisms drive this pathology [33, 34], although it is also possible that by the time of diagnosis the excitable split hand has disappeared.

It would appear that excitability changes occur early, and, by the time of investigation, all nerves appear equally affected. In keeping with excitability changing before motor unit loss, our exploratory analysis found that superexcitability was often abnormal, even when motor unit number was deemed to be “normal” and when EMG data (e.g., cervical segment or FDI‐only) was Gold Coast negative. Consistent with this, Kanai et al., demonstrated significant alterations in excitability in ALS patients with normal CMAP amplitudes [6]. Presymptomatic studies, including clinically unaffected limbs, would be required to further clarify, but it appears peripheral hyperexcitability might, like its cortical equivalent, be an early marker of disease.

We had one patient with a final diagnosis of PLS which we included in the non‐ALS group. This was the Stikvoort Garcia et al. [8] approach, which we kept in order to make our analysis comparable. Interestingly, that patient was predicted as ALS in all models. We also had *n* = 3 patients who transformed within 1 year. Of these, only one (a purely bulbar disease patient when seen) was not predicted as ALS. Stikvoort Garcia et al. also reported cases were re‐classified as ALS on the basis of follow‐up and so may have also had similar predictions. Further work will be required to ascertain if NET can be a useful predictor of ALS transformation.

As a single center study, there will be some selection bias in the cases put forward for investigation. Notwithstanding this, the cases within the non‐ALS group appear similar to those reported by others [8, 17]. As noted earlier, we included patients on medications which could potentially alter excitability and nerve entrapments, which is different from many other studies examining excitability‐based phenomena [5, 8, 35]. However, given that one would hypothesize a negative effect upon diagnostic performance, the preserved performance metrics suggest that the diagnostic potential of excitability indices is robust. In the future, it would be interesting to explore the correlation of quantitative muscle strength (e.g., dynamometry) and symptom duration for hand weakness to better characterize the association between NET and symptoms/clinical signs.

In summary, NET does not demonstrate the split hand phenomenon at the time of ALS diagnosis. Changes to recovery cycle parameters appear to occur with and potentially before motor unit loss. When combined with motor unit parameters from the lateral hand muscles, the data have diagnostic potential.

## Author Contributions

**Alana K. O. Hodgson:** investigation, writing – review and editing. **Christopher J. McDermott:** resources, writing – review and editing. **Pamela J. Shaw:** resources, writing – review and editing. **James J. P. Alix:** conceptualization, methodology, investigation, formal analysis, resources, data curation, writing – original draft, project administration.

## Ethics Statement

We confirm that we have read the Journal's position on issues involved in ethical publication and affirm that this report is consistent with those guidelines.

## Conflicts of Interest

The authors declare no conflicts of interest.

## Supporting information


**Figure S1:** Comparison of superexcitability in ALS patients with/without neurophysiological evidence of the split hand.

**Data S1:** mus70239‐sup‐0002‐Supinfo.docx.

**Table S1:** Nerve excitability parameters across the three muscles.
**Table S2:** Motor unit numbers are significantly reduced across all muscles, motor unit size is significantly increased in APB and FDI.
**Table S3:** The regression parameters when combing APB and FDI data.

## Data Availability

The data that support the findings of this study are available from the corresponding author upon reasonable request.
