# Supplementary figures and images for: The Diagnostic Potential of Axon Excitability Is Consistent Across Hand Muscles in Amyotrophic Lateral Sclerosis
Source: Muscle Nerve. 2026 Apr 11;73(6):1138–45. doi: 10.1002/mus.70239 (PMC13138366; doi:10.1002/mus.70239)

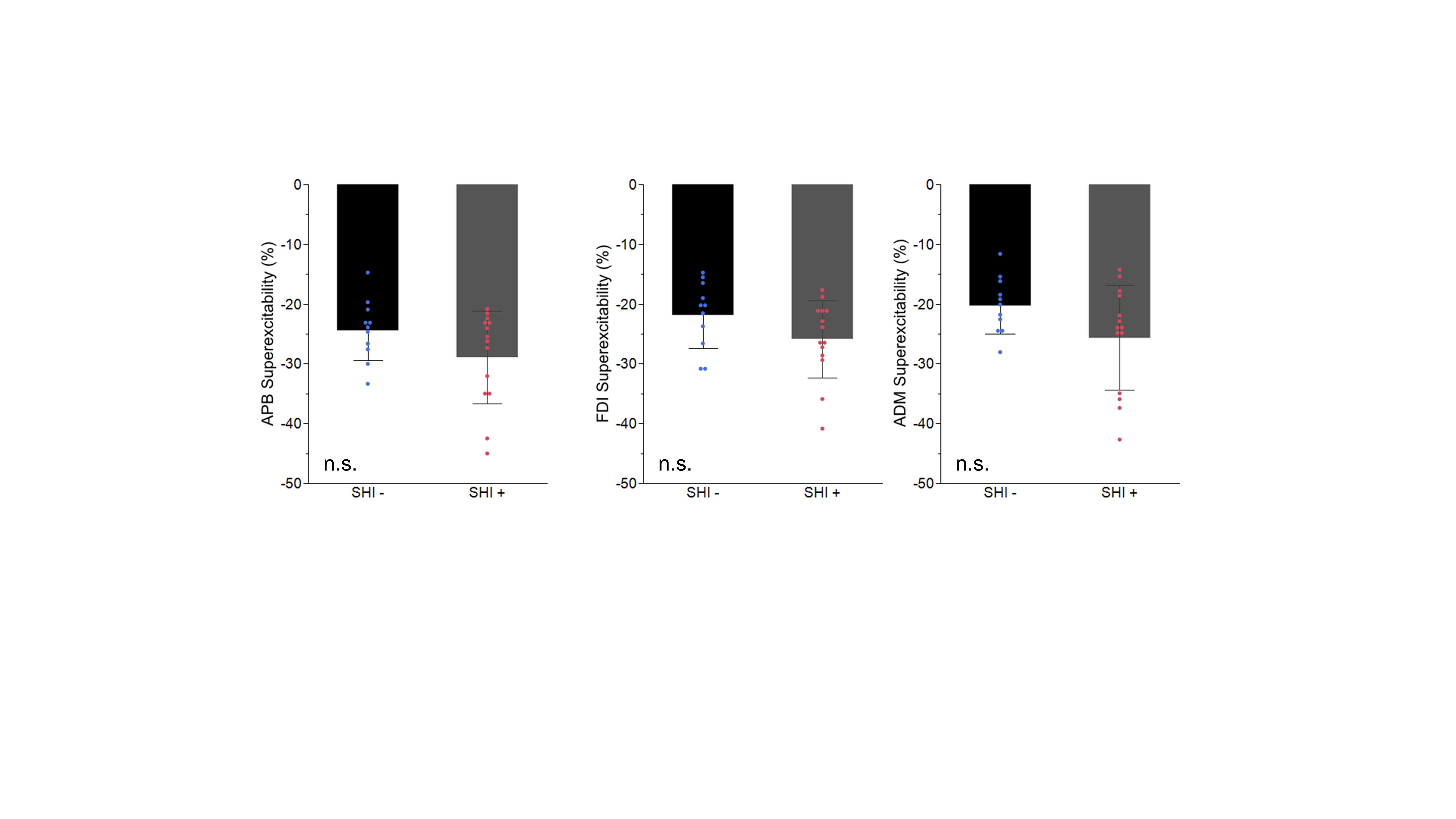

Supplement: Supplementary file 1 — Figure S1: Comparison of superexcitability in ALS patients with/without neurophysiological evidence of the split hand. [file MUS-73-1138-s002.jpg]
