# Supplementary material for: The Diagnostic Potential of Axon Excitability Is Consistent Across Hand Muscles in Amyotrophic Lateral Sclerosis
Source: Muscle Nerve. 2026 Apr 11;73(6):1138–45. doi: 10.1002/mus.70239 (PMC13138366; doi:10.1002/mus.70239)
Supplement: Supplementary file 2 — Data S1: mus70239‐sup‐0002‐Supinfo.docx. [file MUS-73-1138-s001.docx]

**Supplemental figure 1. Comparison of superexcitability in ALS patients with/without neurophysiological evidence of the split hand.**

Patients with ALS were separated into two groups on the basis of their CMAP split hand index measurement (cut-off 5.2, as per ^1^). No significant differences were observed in patients with evidence of a neurophysiological split hand (SHI+) versus those without (SHI-).

**Reference**

1. Menon P, Kiernan MC, Yiannikas C, Stroud J, Vucic S. Split-hand index for the diagnosis of amyotrophic lateral sclerosis. Clin Neurophysiol 2013;124(2):410-416.
