# Supplementary material for: The Diagnostic Potential of Axon Excitability Is Consistent Across Hand Muscles in Amyotrophic Lateral Sclerosis
Source: Muscle Nerve. 2026 Apr 11;73(6):1138–45. doi: 10.1002/mus.70239 (PMC13138366; doi:10.1002/mus.70239)
Supplement: Supplementary file 3 — Table S1: Nerve excitability parameters across the three muscles. Table S2: Motor unit numbers are significantly reduced across all muscles, motor unit size is significantly increased in APB and FDI. Table S3: The regression parameters when combing APB and FDI data. [file MUS-73-1138-s003.docx]

**Supplemental tables**

**Supplemental table 1. Nerve excitability parameters across the three muscles.**

|  | **MEDIAN APB** | | | **ULNAR FDI** | | | **ULNAR ADM** | | |
| --- | --- | --- | --- | --- | --- | --- | --- | --- | --- |
|  | ALS | Non-ALS | p | ALS | Non-ALS | p | ALS | Non-ALS | p |
| **SDTC (ms)** | 0.545 ±0.03 | 0.540 ±0.03 | 0.9 | 0.460 ±0.02 | 0.460 ±0.02 | 0.9 | 0.460 ±0.02 | 0.444 ±0.2 | 0.7 |
| **Rheobase (mA)** | 3.76 ±0.28 | 3.66 ±0.28 | 0.9 | 6.70 ±0.55 | 6.83 ±0.60 | 0.9 | 5.76 ±0.3 | 6.45 ±0.74 | 0.7 |
| **S-R slope** | 4.78 ±0.57 | 4.38 ±0.23 | 0.9 | 3.39 ±0.26 | 3.39 ±0.24 | 0.9 | 3.79 ±0.23 | 3.90 ±0.15 | 0.7 |
| **TEd(10-20ms)** | 69.91 ±1.20 | 66.29 ±1.17 | 0.1 | 64.76 ±1.02 | 62.82 ±0.98 | 0.2 | 65.00 ±1.09 | 62.90 ±1.32 | 0.3 |
| **TEd(90-100ms)** | 48.40 ±1.07 | 44.62 ±0.85 | 0.05 | 45.64 ±0.85 | 46.62 ±1.01 | 0.2 | 48.45 ±1.55 | 44.30 ±1.12 | 0.08 |
| **TEh(10-20ms)** | -77.83 ±1.34 | -75.77 ±2.01 | 0.5 | -74.44 ±1.08 | -70.67 ±1.20 | 0.1 | -72.54 ±1.17 | -68.94 ±1.21 | 0.08 |
| **TEh(90-100ms)** | -126.59 ±3.88 | -118.81 ±3.99 | 0.2 | -122.84 ±3.95 | -112.46 ±3.63 | 0.2 | -125.84 ± 3.10 | -115.88 ± 3.70 | 0.08 |
| **TEh (oversh)** | 14.80 ±0.93 | 14.24 ±0.81 | 0.8 | 11.71 ±1.11 | 10.92 ±0.70 | 0.6 | 10.08 ±0.89 | 9.61 ±0.77 | 0.7 |
| **S2 accomm.** | 20.45 ±0.85 | 20.64 ±0.69 | 0.9 | 19.11 ±0.99 | 18.27 ±0.58 | 0.6 | 17.20 ±1.38 | 17.98 ±0.61 | 0.7 |
| **Resting I/V slope** | **0.56 ±0.02** | **0.63 ±0.02** | **0.03** | **0.57 ±0.02** | **0.65 ±0.02** | **0.02** | 0.55 ±0.02 | 0.62 ±0.03 | 0.1 |
| **Min. I/V slope** | 0.26 ±0.01 | 0.26 ±0.01 | 0.9 | 0.26 ±0.01 | 0.25 ±0.01 | 0.6 | 0.24 ±0.01 | 0.24 ±0.01 | 0.9 |
| **Hyperpol. I/V slope** | 0.36 ±0.01 | 0.38 ±0.01 | 0.5 | 0.38 ±0.01 | 0.37 ±0.01 | 0.6 | 0.35 ±0.01 | 0.36 ±0.2 | 0.7 |
| **RRP (ms)** | **2.77± 0.07** | **3.01 ±0.06** | **0.03** | **2.7 ±0.06** | **3.0 ±0.08** | **0.005** | **2.73 ±0.05** | **2.96 ±0.07** | **0.01** |
| **Refract. 2.5ms)** | 13.4 ±3.8 | 20.6 ±2.9 | 0.2 | 9.0 ±3.5 | 16.7 ±2.5 | 0.1 | **7.48 ±1.95** | **14.51 ±2.26** | **0.03** |
| **Superex. (%)** | **-26.94 ±** **1.40** | **-19.65 ± 1.22** | **0.001** | **-24.1 ±** **1.3** | **-15.8 ±** **1.3** | **0.0001** | **-23.30 ±1.53** | **-15.91 ±1.22** | **0.002** |
| **Subex. (%)** | 13.86 ±0.86 | 14.24 ±0.89 | 0.8 | 11.0 ±1.2 | 10.5 ±1.1 | 0.8 | 9.09 ±1.05 | 9.05 ±0.65 | 0.9 |

SDTC – strength duration time constant, S-R slope – stimulus response slope, TEd – threshold reductions to depolarising currents, TEh – threshold reductions to hyperpolarising currents, TEh(oversh) – TEh(overshoot), S2 accomm. – S2 accommodation, I/V – current/voltage, RRP – relative refractory period, ms – milliseconds. Refract. – Refractoriness, Superex. – Superexcitability, Subex. – Subexcitability. Data are presented as mean ± standard error of the mean. Significant group differences are shown in bold. Similar parameters were grouped together for FDR correction, these groupings are denoted by the shading/no shading.

**Supplemental table 2. Motor unit numbers are significantly reduced across all muscles, motor unit size is significantly increased in APB and FDI.**

|  | ***MEDIAN APB*** | | | ***ULNAR FDI*** | | | ***ULNAR ADM*** | | | |
| --- | --- | --- | --- | --- | --- | --- | --- | --- | --- | --- |
|  | *ALS* | *Non-ALS* | *p* | *ALS* | *Non-ALS* | *p* | *ALS* | *Non-ALS* | *p* |  |
| **Motor unit number** | **53.6**  **±6.5** | **92.7**  **±8.9** | **0.003** | **54**  **±5.7** | **100**  **±8.3** | **8.2e-5** | **62.1**  **±7.0** | **90.3**  **±7.5** | **0.02** |  |
| **Motor unit amp. (µV)** | **172.**  **5±15.7** | **115.5**  **±9.4** | **0.005** | **145.3**  **±9.5** | **113.2**  **±11.1** | **0.03** | 155.9  ±19.4 | 113.3  ±93.4 | 0.06 |  |
| **CMAP amp. (mV)** | **7.6 ±0.6** | **9.4 ±0.7** | **0.03** | **7.9 ±0.8** | **11.1 ±0.8** | **0.01** | **7.5 ±0.5** | **9.1 ±0.5** | **0.03** |  |

Significant group differences are shown in bold. amp = amplitude.

**Supplemental table 3. The regression parameters when combing APB and FDI data.**

| **Term** | **Estimate** | **Std Error** | **Chi Sq.** | **Prob >Chi sq** | **Prediction formula** |
| --- | --- | --- | --- | --- | --- |
| Intercept | 1.13268182 | 4.6505532 | 0.06 | 0.8076 | -1.132681818  +0.0389454296*APB motor unit size  +0.0916571115*APB TEh(90-100ms)  +-0.069421663*FDI motor unit number  +-0.661916852*FDI superexcitability |
| APB motor unit size | -0.0389454 | 0.0165394 | 5.54 | 0.0185 |  |
| APB TEh(90-100ms) | -0.0916571 | 0.0429719 | 4.55 | 0.0329 |  |
| FDI motor unit number | 0.06942166 | 0.0313053 | 4.92 | 0.0266 |  |
| FDI Superexcitability (%) | 0.66191685 | 0.2402011 | 7.59 | 0.0059 |  |
